# Exhausted and Apoptotic BALF T Cells in Proinflammatory Airway Milieu at Acute Phase of Severe Mycoplasma Pneumoniae Pneumonia in Children

**DOI:** 10.3389/fimmu.2021.760488

**Published:** 2022-01-17

**Authors:** Xi Chen, Fang Liu, Baoying Zheng, Xiaohui Kang, Xiaolin Wang, Wenjun Mou, Hui Zhang, Anxia Jiao, Shunying Zhao, Jingang Gui

**Affiliations:** ^1^ Laboratory of Tumor Immunology, Beijing Pediatric Research Institute, Beijing Children’s Hospital affiliated to the Capital Medical University, National Center for Children’s Health, Beijing, China; ^2^ Department of Interventional Pulmonology, Beijing Children’s Hospital affiliated to the Capital Medical University, National Clinical Research Center for Respiratory Diseases, National Center for Children’s Health, Beijing, China; ^3^ Department of Pulmonology, The Children’s Hospital Affiliated to the Capital Institute of Pediatrics, Beijing, China; ^4^ Department of Respiratory Diseases, Beijing Children’s Hospital affiliated to the Capital Medical University, National Clinical Research Center for Respiratory Diseases, National Center for Children’s Health, Beijing, China

**Keywords:** mycoplasma pneumoniae pneumonia (MPP), children, bronchoalveolar lavage fluid (BALF), T cells, proinflammatory milieu, cell exhaustion

## Abstract

Severe mycoplasma pneumoniae pneumonia (MPP) in children presents with serious clinical complications. Without proper and prompt intervention, it could lead to deadly consequences. Dynamics of the inflammatory airway milieu and activation status of immune cells were believed to be the hallmark of the pathogenesis and progress of the disease. In this study, by employing the T-cell sorting and mRNA microarray, we were able to define the main feature of the chemokine/cytokine expression and the unique characteristics of T cells in the bronchoalveolar lavage fluid (BALF) from severe MPP patients at acute phase. Our study for the first time delineated the molecular changes in isolated BALF T cells in severe MPP children with respect to the cytokine/chemokine expression, cell activation, exhaustion, and apoptosis. By comparing the BALF aqueous expression of cytokines/chemokines with that in sorted T cells, our data give a preliminary clue capable of finishing out the possible cell source of the proinflammatory cytokines/chemokines from the BALF mixture. Meanwhile, our data provide a distinctively pellucid expression profile particularly belonging to the isolated BALF T cells demonstrating that in the inflammatory airway, overactivated T cells were exhausted and on the verge of apoptotic progress.

## Introduction

Mycoplasma pneumoniae (Mp) pneumonia (MPP) is one of the most common community-acquired diseases in children. In most occasions, it leads to mild symptoms that are normally self-limited. However, in the context of immunodeficiency or other collateral infections, Mp infection could cause severe complications including bronchiolitis obliterans, fulminant inflammatory diseases, encephalitis, and even life-threatening acute respiratory distress syndrome. Particularly, in recent years, along with the increasing frequency of macrolide-resistant cases, it is not uncommon to see more and more severe cases ascribed solely to Mp infection (1). It is well accepted that Mp infection induces proinflammatory cytokines and chemokines release in respiratory tract and activates variety of immune cells (2–6). Other than the concurrent increase in proinflammatory cytokines such as IL-8 and TNF-α in BALF, there were enough evidence for the critical role of T cells in the pathogenesis of severe MPP. Particularly, T-cell activation and cell-mediated inflammatory damage together with cytokine-directed proinflammatory milieu in respiratory tract were believed to be key components in the progression of MPP (7–10). However, all these studies were based on the bronchoalveolar lavage fluid (BALF) sample that mainly comprised miscellaneous cells and cytokines/chemokines of undefined origin. In this sense, it is impossible to know the exact behavior of activated T cells in BALF and how they contribute to the inflammatory milieu during Mp infection without purifying the T cells. In the present study, the major cell compartments in BALF were compared between MPP patients at acute phase and children recovered from aspiration of respiratory foreign body (FB). The cytokine and chemokine levels in BALF and serum were also compared. Differentially expressed genes (DEGs) in BALF cells and FB controls were determined by mRNA microarray. Through comparing the DEGs in BALF cells and those in sorter-purified BALF CD3+ T cells from these two groups, we were able to pinpoint the T-cell or non-T-cell origin of critical cytokines/chemokines as inflammatory mediators. In addition, by analyzing the DEGs related to particular T-cell functions in patients and FB controls, we found that cell exhaustion and apoptosis programing were initialized in the overactivated BALF CD3+ T cells in MPP children. Results from our study would pave the road for a better understanding for the complicated proinflammatory airway microenvironment intertwined with multiple immune cells and cytokines.

## Materials and Methods

### Study Population

All the patients involved in this study were children being newly diagnosed as severe MPP at acute phase and admitted to the Department of Respiratory Disease, Beijing Children’s Hospital or The Children’s Hospital Affiliated to the Capital Institute of Pediatrics. The controls in this study were children subjected to aspiration of foreign body (FB) out of the airway or healthy children for medical examination. Severe MPP diagnoses were based on the clinical presentations including cough, fever, dry or productive sputum, dyspnea, abnormal breath sounds, as well as chest radiographs. *M. pneumoniae* infection was confirmed by ELISA serum MP IgM test, and sputum MP DNA (>10^4^ copy/L) load was determined by real-time quantitative PCR. Cases of MPP coinfected with other microorganisms were excluded from this study. Among these subjects, cell pellets from BALF of 13 patients (7 males and 6 females, average age 6.21 ± 2.72) and 13 sex-matched FB controls (7 males and 6 females, average age 2 ± 2.82) were taken for cell subpopulation analysis by flow cytometry. Upper aqueous phase of BALF from 48 (25 males and 23 females, average age 7.19 ± 3.23 years) patients and 30 FB controls (18 males and 12 females, average age 1.62 ± 1.75 years) were used for cytokine/chemokine concentration determination with a Luminex 200 system. Serum drawn from 20 patients (10 males and 10 females, average age 6.6 ± 3.28) and 20 age- and sex-matched healthy controls (HC) (10 males and 10 females, average age 6.65 ± 3.17) were used for cytokine/chemokine determination by Luminex 200. PBMCs isolated from blood from 5 severe MPP patients and 6 FB controls were stained with CD3 and CD45RA/CD45RO for naive and memory T-cell determination. BALF cells from 4 severe MPP patients and 5 FB controls were used for annexin V and JC-1 staining to determine the apoptosis rate. BALF cells from BALF cell pellets from 3 severe MPP patients and 3 FB controls were collected for mRNA microarray chips. BALF T cells sorted from 3 severe MPP patients and 3 FB controls (>98% purity) were used for mRNA microarray assays. Basic information for MPP patients and FB controls and how these clinical samples are being used are summarized in **Table 1**. The research purpose and consent were informed and duly signed by patients or their parents/legal guardians. Protocols and procedures in this study were approved by the Ethics Committee of Beijing Children’s Hospital and the Ethics Committee of The Children’s Hospital Affiliated to the Capital Institute of Pediatrics.

**Table 1:** Patient Information and each experiment involved.

Experiment involved	Group	*N*	Male: Female	Age (year, mean ± SE)
**Total**	FB (control)	67	40:27	3.54 ± 3.36
	MPP	85	41:44	6.90 ± 3.18
**Cell surface marker staining**	FB	13	7:6	2.00 ± 2.82
	MPP	13	7:6	6.21 ± 2.72
**Intracellular cell staining**	FB	11	8:3	1.80 ± 1.48
	MPP	11	4:7	7.00 ± 3.29
**Cytokines/chemokines (aqueous BALF)**	FB	30	18:12	1.62 ± 1.75
	MPP	48	25:23	7.19 ± 3.23
**Cytokines/chemokines (blood)**	FB	20	10:10	6.65 ± 3.17
	MPP	20	10:10	6.60 ± 3.28
**mRNA microarray (BALF cells)**	FB	3	2:1	2.00 ± 1.73
	MPP	3	1:2	4.67 ± 2.52
**mRNA microarray (sorted BALF T cells)**	FB	3	2:1	2.33 ± 2.31
	MPP	3	1:2	6.33 ± 2.08

FB, foreign body removal control; MPP, Mp infection patient.

### Flow Cytometry Analysis of BALF

Cells in BALF were pelleted by centrifugation at 600×*g* for 5 min at RT. Supernatant was then collected and stored at −80°C for cytokine determination. BALF cells were then purified with Ficoll-Hypaque Solution following with a centrifugation of 1,000×*g* for 15 min. The cell pellets were washed with phosphate-buffered saline (PBS) twice and stained for flow cytometry analysis. After staining for 20 min, cells were analyzed using BD FACSCalibur Flow Cytometer (Becton Dickinson, San Jose, CA, USA). Lymphocyte gating was performed according to the forward scatter and side scatter signal. The following mouse anti-human monoclonal antibodies were used for labeling BALF cells: fluorescein isothiocyanate (FITC)-conjugated anti-CD3, anti-CD8, anti-CD11b, anti-TCRαβ, anti-HLA-DP, DQ, DR, and anti-CD56; phycoerythrin (PE)-conjugated anti-CD45, anti-CD45RA, anti-CD14, anti-CD19, anti-CD103, and anti-CD25; peridinin chlorophyll protein (PerCP)-conjugated anti-CD3, anti-CD16, anti-CD45, and anti-CD45RO; and allophycocyanin (APC)-conjugated anti-CD3, anti-CD4, anti-CD45, anti-TCRγδ, anti-HLA-DR, anti-IFN-γ, anti-IL-2, and anti-TNF-α. For apoptosis analysis, BALF cells were stained with FITC-anti-CD3, followed by APC-annexin V in calcium containing binding buffer (0.1 M Tris, pH 7.4, 1.5 M NaCl, 25 mM CaCl_2_). The JC-1 detection assay was performed to measure the mitochondrial membrane potential of T cells in BALF. In brief, BALF cells were stained with APC-CD3, followed by 2.5 μg/ml JC-1 in prewarmed medium at 37°C in the dark for 30 min. The JC-1 fluorescence shift was used to evaluate the cell mitochondrial membrane potential change. For cell exhaustion marker detection, FITC-anti-PD1, PE-anti-TIM3, and PeCy7-anti-TIGIT were used. In some experiments, blood samples were used for determining CD45RA versus CD45RO expression in CD3+ T cells. Data were analyzed using FlowJo 7.6 software (Treestar, Ashland, OR, USA).

### Multiplex Cytokine Analysis

A multiplex assay for quantitative determination of inflammatory mediators was applied to assess concentrations of selected cytokines in BALF and blood serum. Fourteen cytokines (IFN-α2, IFN-γ, IL-12p70, IL-15, IL-17a, IL1-β, IL-2, IL-4, IL-6, IL-8, IL-10, MCP-1, RANTES, TNF-α) were analyzed using Human Cytokine/Chemokine Magnetic Bead Panel 96 Well Plate Assay (EMD Millipore, Burlington, MA, USA). The assay was run according to the manufacturer’s protocol. Plates were read on a MILLIPLEX Analyzer system and analyzed using MILLIPLEX Analyst software (Millipore Laboratories). A 5-parameter model was used to calculate the final concentration of each cytokine based on the standard curve generated along with each test.

### BALF Cells and Sorted T Cell mRNA Microarray Analysis

For mRNA expression measurement in total BALF cells or sorted BALF T cells, cells were pelleted down by centrifugation and directly lysed in Trizol. For purification of CD3+ T cells from BALF, BALF cells were stained with FITC-conjugated anti-CD3 antibody and CD3+ T cells were sorted with a BD Aria II cell sorter. About 5 × 10^5^ CD3+ T cells were collected from each sample and lysed in Trizol. The mRNA was extracted according to the Trizol protocol and quantified with Nano-Drop. Agilent SurePrint G3 Human Gene Expression v3 microarray (8*60K, design ID: 072363) was applied for BALF cell samples. The mRNA from sorted BALF T cells was amplified by linear amplification using GeneChip™ Whole Transcript (WT) Pico Reagent Kit followed by mRNA quantitative hybridization assay. After hybridization, box line diagram analysis, scatter diagram analysis, and principal component analysis (PCA) were performed to analyze the experimental image. DEGs were selected based on the change of expression >2 fold (*p* < 0.05), splicing index <−2 or >2, Exon-level of *p*-value <0.05, as well as function belonging to our study of interest (chemokine, cytokine, T-cell activation and exhaustion, and cell apoptosis). Selected DEGs were then subjected to cluster analysis, GO analysis, and KEGG pathway analysis.

### Statistical Analysis

Data in this study were expressed as mean ± SEM and were processed by GraphPad Prism 8 software. Student’s *t-*test was performed to test significant difference between experiment groups. Statistical significances were represented as ^*^ *p* < 0.05; ^**^ *p* < 0.01; ^***^ *p* < 0.001.

## Results

### Change in T-Cell Subpopulation in MPP BALF With an Enhanced Activation, Exhaustion, and Apoptosis

**Figure 1:**
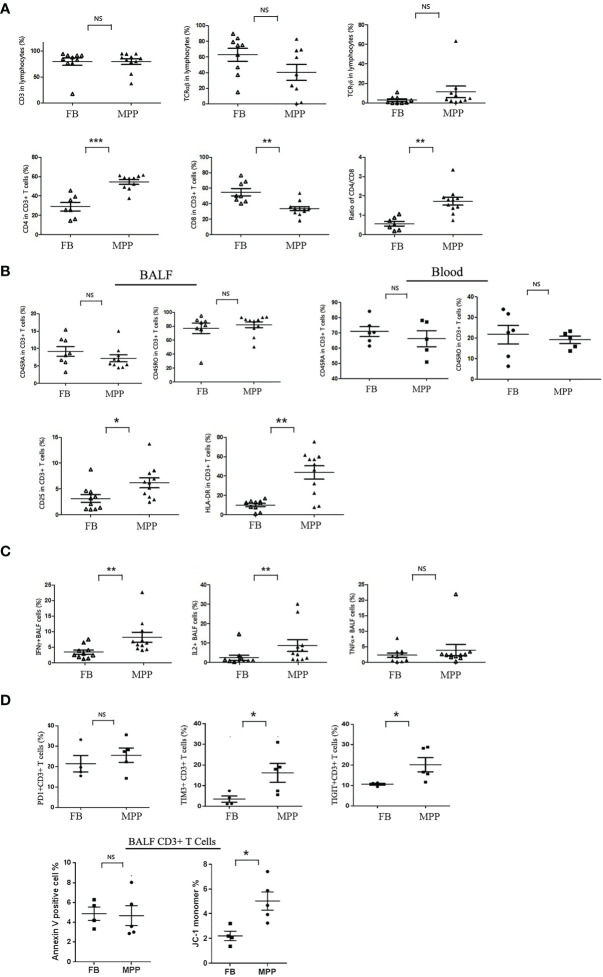
BALF cells from patients (MPP) and controls (FB) were stained with monoclonal antibodies conjugated with flourochrome. Cells were washed with PBS and then subjected to the analysis by a FACScalibur flow cytometry. Data were processed with Flowjo software. Every dot in the scatter plot indicates a patient or control sample. ^*^ *p* < 0.05; ^**^ *p* < 0.01; ^***^ *p* < 0.001; NS, no significant difference. **(A)** T-cell subgroups and CD4/8 ratio were represented as scatter plots. **(B)** Cytometric analysis of naive (CD45RA+) vs. memory (CD45RO+) in BALF and blood CD3+ cells, as well as the activation status (CD25+ and HLA-DR+) of BALF T cells (gating CD3+ population). **(C)** BALF cells were stimulated with TCR ligation by anti-CD3/CD28 in the presence of Golgi Stop. Intracellular cytokines (IFN-γ, IL-2, and TNF-α) expression was detected with corresponding flourochrome-conjugated antibody following cell perforation. **(D)** BALF cells from MPP patients and FB controls were stained with PD1, TIM3, and TIGIT; their ratio in CD3+ T-cell gate was represented (upper panels). BALF cells from MPP patients and FB controls were stained with annexin V and JC-1 for apoptosis analysis. The ratios of annexin V-positive cells and JC-1 monomer + cells in CD3+ T-cell gate were represented as dot plot.

Clarifying what are the major cell compartments changed in BALF of pediatric severe MPP patients would infer the major cell-mediated events in the proinflammatory airway. With this aim, we stained the BALF cells harvested from Ficoll-Hypaque solution separation with a plethora of fluorochrome-conjugated antibodies identifying the major immune cell populations and analyzed by flow cytometry. The results found no evidence for a difference in the distribution of major cell compartments in BALF from severe MPP patients and that in FB controls (BALF from patients subjected to foreign body removal). The flow cytometry data indicated that CD3+ T cells were the main subpopulations accounting for about 85% of the mononuclear cells in MPP and FB BALF (mean ± SD: 84.6% ± 11.0% in MMP vs. 86.7 ± 6.4 in control) (**Figure 1A**). B cells, by contrast, presented with a mean ratio of less than 3% of the BALF mononuclear cells (2.7% ± 1.4% in MPP vs. 2.5 ± 2.2 in control). CD16+CD56+ NK cells were shown to comprise 1.7% ± 1.1% in MPP BALF mononuclear cell versus 1.7% ± 2.1% in control. CD14+ macrophages in our experiment displayed a percentage of 7.3% ± 5.0% in MPP BALF versus 5.0% ± 3.8% in control BALF (**Supplementary Figure S1B**). While there was a consistent increase in the percentage of blood neutrophils in MPP patients compared with that in FB (52.42 ± 1.98 in MPP vs. 36.62 ± 2.26 in FB, *p* < 0.001), the percentages of neutrophils were quite variable in both unpurified MPP and FB BALF (**Supplementary Figure S1B**). The major cell distribution in BALF and the gating strategy were illustrated in **Supplementary Figure S1A**.

Now that we knew the major cell population in BALF mononuclear cells was CD3+ T cells, we were interested in the distribution of the CD4 and CD8 subpopulations in T cells. Our data indicated that the percentage of CD4+ T cells was remarkably increased while the ratio of CD8+ T cells was greatly decreased in CD3+ T cells of MPP BALF (**Figure 1A**). This led to a CD4/CD8 ratio shift in MPP BALF compared with that in control BALF (mean ± SE: 1.7 ± 0.2 in MPP vs. 0.6 ± 0.1 in FB). We then determined the naive versus memory status of these T cells in BALF. To our surprise, no matter in MPP patients or in FB controls, majority of CD3+ T cells in BALF were expressing CD45RO molecule, a memory cell marker. This was in contrast to what is in the PBMCs of children’s blood in which majority of T cells express CD45RA-naive T-cell marker (**Figure 1B**). In addition, through scanning multiple surface markers in CD3+ T cells, we proved that BALF CD3+ T cells in MPP patients exhibited an enhanced activation as expression of CD25 (IL-2 receptor α chain) and HLA-DR was significantly upregulated (**Figure 1B**). Other markers facilitating T-cell activation and cell adhesion such as CD127 (IL-7 receptor α chain) and CD103 (a mucosal residential memory T-cell marker) were not disturbed relative to the levels in FB BALF T cells. The enhanced activation of MPP BALF T cells was double confirmed when we stimulated the BALF T cells in a *petri dish* with anti-CD3/CD28 antibodies. Upon TCR ligation, the MPP BALF T cells produced more IFN-γ and IL-2 than those from control BALF (**Figure 1C**). Surprisingly, TNF-α release was at similar level between patients and control BALF T cells in response to TCR stimulation.

To further characterize the activated BALF T cells in MPP patients, we measured the expression of cell exhaustion markers and determined the cell apoptosis of the BALF CD3+ T cells. As shown in **Figure 1D**, compared with FB controls, BALF CD3+ T cells from MPP patients exhibited an increased expression in cell surface exhaustion makers TIM3 and TIGIT but not PD1. While there is no difference in annexin V staining between patients and control BALF T cells, CD3+ T cells in MPP BALF had a higher percentage of JC-1 monomer signal indicating an altered mitochondria membrane potential. These data suggested that CD3+T cells in MPP patient BALF were exhausted with more apoptotic events initialized.

### Specific Cytokine Profile in MPP BALF and Serum

**Figure 2:**
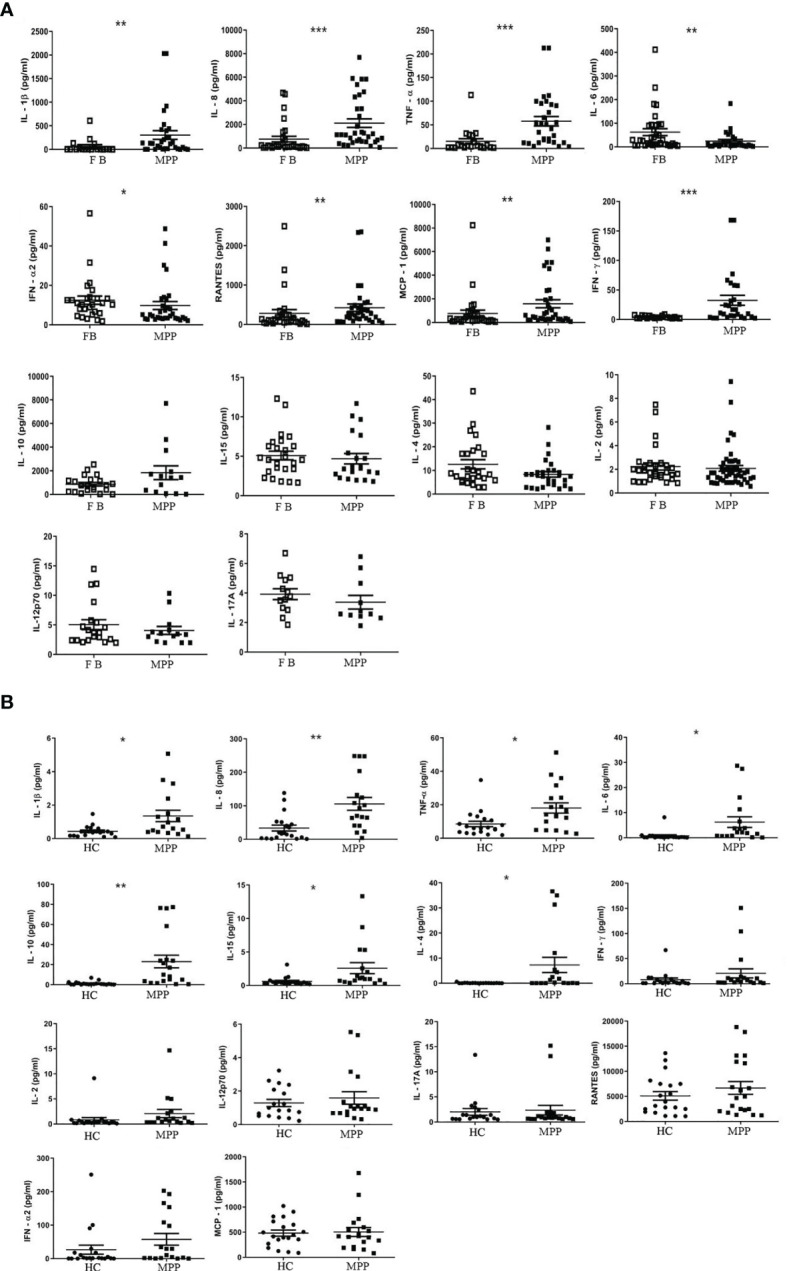
The absolute concentration (pg/ml) of each indicated cytokine in **(A)** aqueous phase of BALF or in **(B)** serum was determined *via* Luminex xMAP technology by a calculation based on a standard curve generated from the quantification standards of each cytokine the manufacture provided. Student’s *t*-test was employed to analyze the difference of the cytokine concentration in **(A)** BALF between patient group (MPP) and control group (FB) and **(B)** in serum between patient (MPP) and control group (HC). ^*^ *p* < 0.05; ^**^ *p* < 0.01; ^***^ *p* < 0.001.

Based on our analysis for the cell compartments in BALF, we boldly believed that the predominant CD3+ T cells in BALF, particularly the increased CD3+CD4+ T cells in MPP BALF, at least partially contributed to the proinflammatory cytokine/chemokine microenvironment. With this curiosity, we measured the concentration of 14 cytokines mediating inflammation. As expected, Luminex data revealed that 6 proinflammatory cytokines (IL-1β, IL-8, TNF-α, IFN-γ, RANTES, MCP-1) out of 14 cytokines in MPP patient BALF presented in a level significantly higher than control BALF. By contrast, IL-6 and IFN-α2 were shown to decrease in MPP BALF. Other cytokines (IL-2, IL-12, IL-17A, IL-10, IL-4, IL-15) in patient BALF were found at similar levels to controls (**Figure 2A**; **Table 2**).

**Table 2:** Cytokine/chemokine expression in BALF and serum.

Upregulation or Downregulation	Cytokines	BALF (pg/ml, mean ± SE)		Serum (pg/ml, mean ± SE)	
		FB	MPP	HC	MPP
In both BALF and serum	IL-1β	64.9 ± 34.2	305.5 ± 91.3^***^ ↑	0.43 ± 0.07	1.35 ± 0.33^*^ ↑
	IL-8	764.7 ± 234	2,040.7 ± 363.1^***^ ↑	33.4 ± 8.7	105.7 ± 18.5^**^ ↑
	TNF-α	15.0 ± 5.6	57.7 ± 9.9^***^ ↑	8.5 ± 1.6	16.1 ± 3.0^*^ ↑
	IL-6	62.6 ± 14.5	24.5 ± 7.0^**^ ↓	0.75 ± 0.41	6.2 ± 0.15^*^ ↑
In BALF only	IFN-γ	4.2 ± 0.4	32.5 ± 8.2^***^ ↑	8.0 ± 3.3	20.7 ± 8.9
	RANTES	280.4 ± 94.8	424.8 ± 91.6^**^ ↑	5,100 ± 817	6,683 ± 1211
	MCP-1	763 ± 278	1,557 ± 335^**^ ↑	482 ± 59	504 ± 87
	IFN-α2	12.5 ± 2.1	9.8 ± 2^*^ ↓	26.7 ± 13.11	57.6 ± 17.0
In serum only	IL-10	864.9 ± 147.3	1,834 ± 562	1.32 ± 0.38	23.2 ± 6.1^**^ ↑
	IL-15	5.1 ± 0.54	4.67 ± 0.64	0.59 ± 0.14	2.6 ± 0.61^*^ ↑
	IL-4	12.6 ± 1.9	8.3 ± 1.1	0.1 ± 0.03	7.3 ± 2.9^*^ ↑
Neither in BALF nor serum	IL-2	2.2 ± 0.3	2.1 ± 0.24	0.83 ± 0.43	2.1 ± 0.8
	IL-12	5.0 ± 0.82	4.1 ± 0.65	1.3 ± 0.2	1.6 ± 0.36
	IL-17A	3.9 ± 0.35	3.4 ± 0.44	2.0 ± 0.65	2.4 ± 0.93

FB, foreign body removal control; MPP, Mp infection patient; HC, healthy control; “↑” upregulation; “↓” downregulation.

^*^p = 0.05; ^**^p = 0.01; ^***^p = 0.001.

We then asked the question if the enhanced proinflammatory cytokine profile in the respiratory track mirrors the cytokine expression in the circulating blood. Our data demonstrated a very different cytokine profile between BALF and serum. The serum concentration of IL-1β, IL-8, and TNF-α, similar to that in BALF, presented with a higher level in MPP patients. In addition, IL-10, IL-4, and IL-15, three cytokines exhibiting no difference in MPP BALF, were found to increase in MPP serum relative to control serum. To our surprise, IL-6, the downregulated cytokine in MPP BALF, was shown to increase in MPP serum compared with that in control (**Figure 2B**; **Table 2**). There was no differential serum expression of other cytokines including IL-2, IL-12, IL-17A, RANTES, MCP-1, and IFN-α2, some of which were higher in patient BALF samples. The distinct cytokine profiles between circulation and airway of severe MPP patients (summarized in **Table 2**) pinpointed a critical role of local immunity in the airway in response to Mp infection. Worth to mention, compared with serum levels, IL-6, IL-8, IL-10, RANTES, and MCP-1 were expressed in a noticeably different concentration range in both MPP and control BALF. This implies that these inflammation mediators are most likely indispensable players in airway immunity during Mp infection.

### Gene Upregulation in Activation, Exhaustion, and Apoptosis of BALF T Cells From MPP Patients

**Figure 3:**
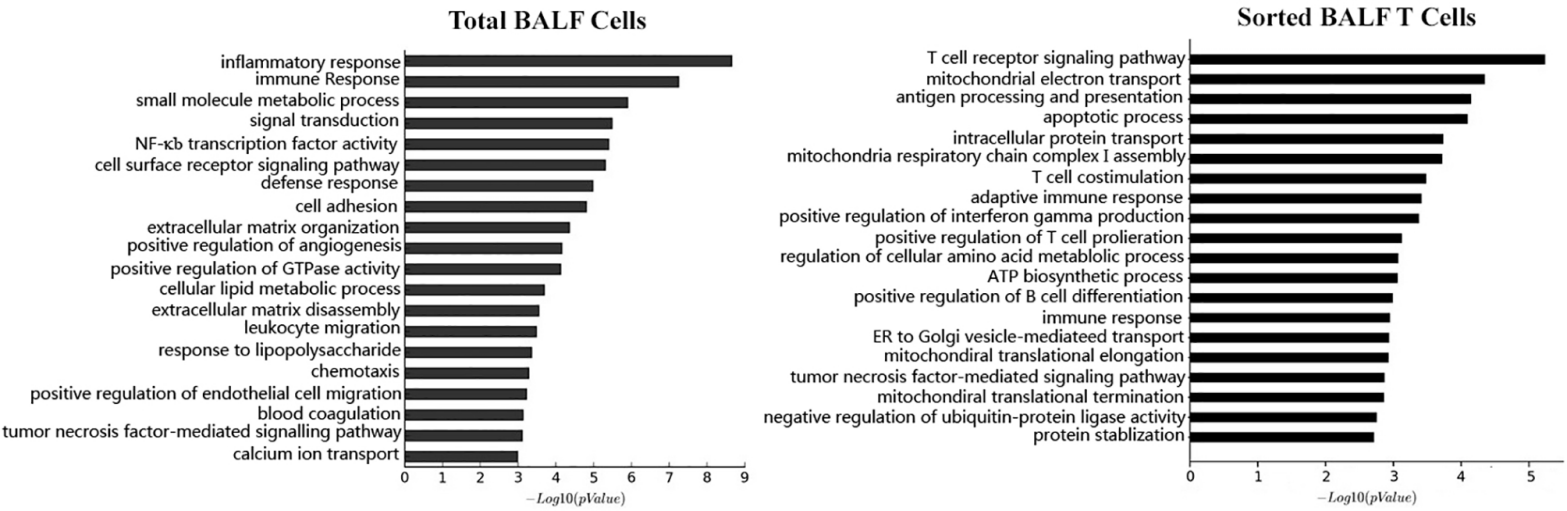
Based on the microarray mRNA chip results, the mRNA expression level were compared between patient BALF samples and FB control samples. Differentially expressed genes (DEGs) in BALF samples were subjected to Gene Ontology (GO) analysis. Cell functions these DEGs involved are categorized and ranked in a sequential order of relevance (upper bar to lower bar is from high to low). The left panel is the result from GO analysis of total BALF cells. The right panel is the result from GO analysis of sorted BALF CD3+ T cells.

With knowledge on the cell components and cytokine profiles in MPP BALF, we were of great curiosity to acquire the information on the gene expression of the BALF cells. Through mRNA microarray analysis, we found 1,866 upregulated genes and 1,590 downregulated genes in BALF cells from refractory MPP patients, compared with those from FB controls. Gene Ontology (GO) analysis clustered these transcriptional differences into the inflammatory response and immune response ranking at the first place and second place, respectively (**Figure 3**, left panel). This was expected because it had been well documented that the inflammation and immune response were major events during Mp infection (9). Theoretically, through comparing the DEGs (obtained from comparison between MPP patients and FB controls) between unpurified BALF cells and sorted T cells, we could dig out the DEGs exclusively presented in BALF T cells from severe MPP patients. With this aim, we sorted patient and FB control CD3+ T cells from BALF and measured the transcriptional expression by mRNA microarray. The enhanced resolution via cell sorting enabled us to disclose the unique gene expression pattern in BALF T cells, which is not approachable from unpurified BALF cells. Our mRNA expression data determined by microarray chips found 8,918 upregulated DEGs and 4,751 downregulated DEGs in sorted T cells from MPP BALF, compared with those T cells sorted from FB control BALF. Gene Ontology analysis revealed that the top changes in these T cells were related to T-cell receptor and costimulation signal, mitochondria respiration, antigen processing and presentation, as well as apoptotic process (**Figure 3**, right panel).

**Figure 4:**
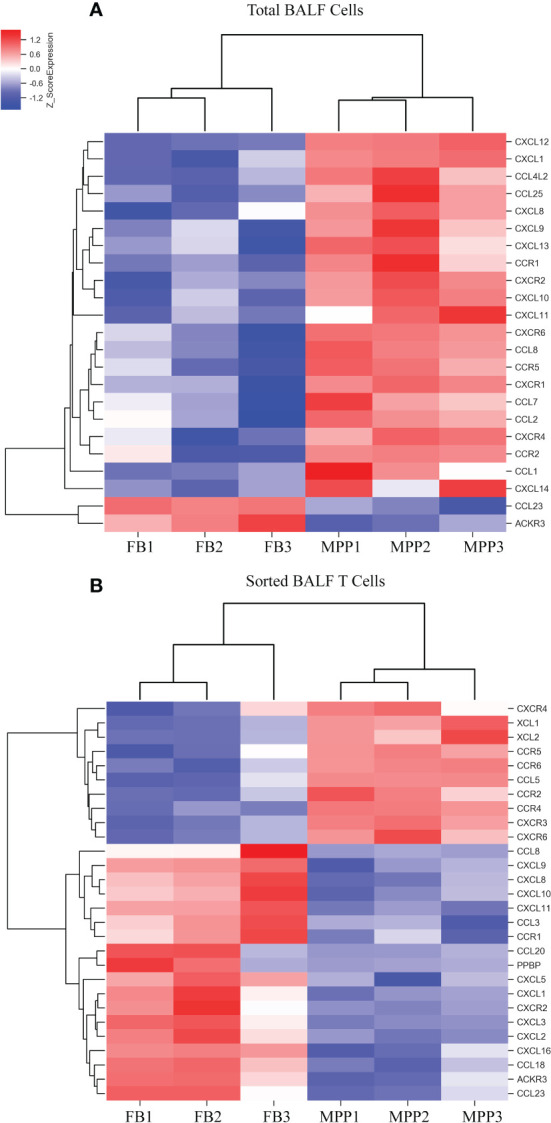
DEGs of chemokines from microarray results were represented as heat maps. Colors indicate relative expression levels. The gradient color from red to blue means expression of the listed gene from upregulation to downregulation. Gene names of DEGs are listed on the right. Similar pattern of expression are clustered closer with linked line. **(A)** The heat map for DEGs of chemokine gene in BALF cells. Each sample represents BALF cells from an individual patient or FB control. **(B)** The heat map for DEGs of chemokine gene in sorted CD3+ T cells. Each sample represents sorted CD3+ T cells from an individual patient or FB control.

In addition, we observed a notable DEG pattern with a remarkable increase in various chemokines in total BALF cells versus that in sorted T cells. The transcriptional expression of a vast number of different CCL and CXCL family chemokines were found to increase markedly in total BALF cells from MPP patients compared with control BALF cells (**Figure 4A**), whereas this drastic change in chemokine expression profile was not observed in sorted patient BALF T cells in comparison with FB controls (**Figure 4B**). Among these, IL-8 (CXCL8), an important proinflammatory factor, was notably increased in patient BALF cells but not in sorted BALF T cells. Some important chemokines were expressed significantly higher in both patient BALF cells and sorted BALF T cells such as CCR5, the receptor for RANTES (CCL5), and CCR2, the receptor for MCP-1. To be noted, chemokines CCR6, XCL1, XCL2 and CCL5 (RANTES) expressed exclusively with a higher level in sorted patient BALF T cells (**Figure 4B**).

**Figure 5:**
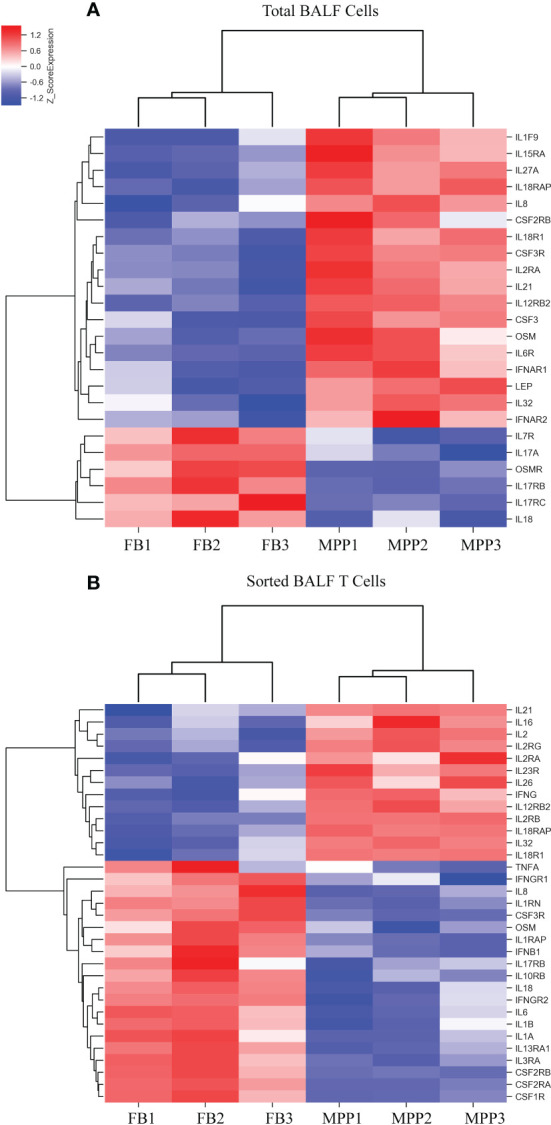
DEGs of cytokines from microarray results were represented as heat maps. Colors indicate relative expression levels. The gradient color from red to blue means expression of the listed gene from upregulation to downregulation. Gene names of DEGs are listed on the right. Similar pattern of expression are clustered closer with linked line. **(A)** The heat map for DEGs of cytokine gene in BALF cells. Each sample represents BALF cells from an individual patient or FB control. **(B)** The heat map for DEGs of cytokine gene in sorted CD3+ T cells. Each sample represents CD3+-sorted cells from an individual patient or FB control.

**Figure 6:**
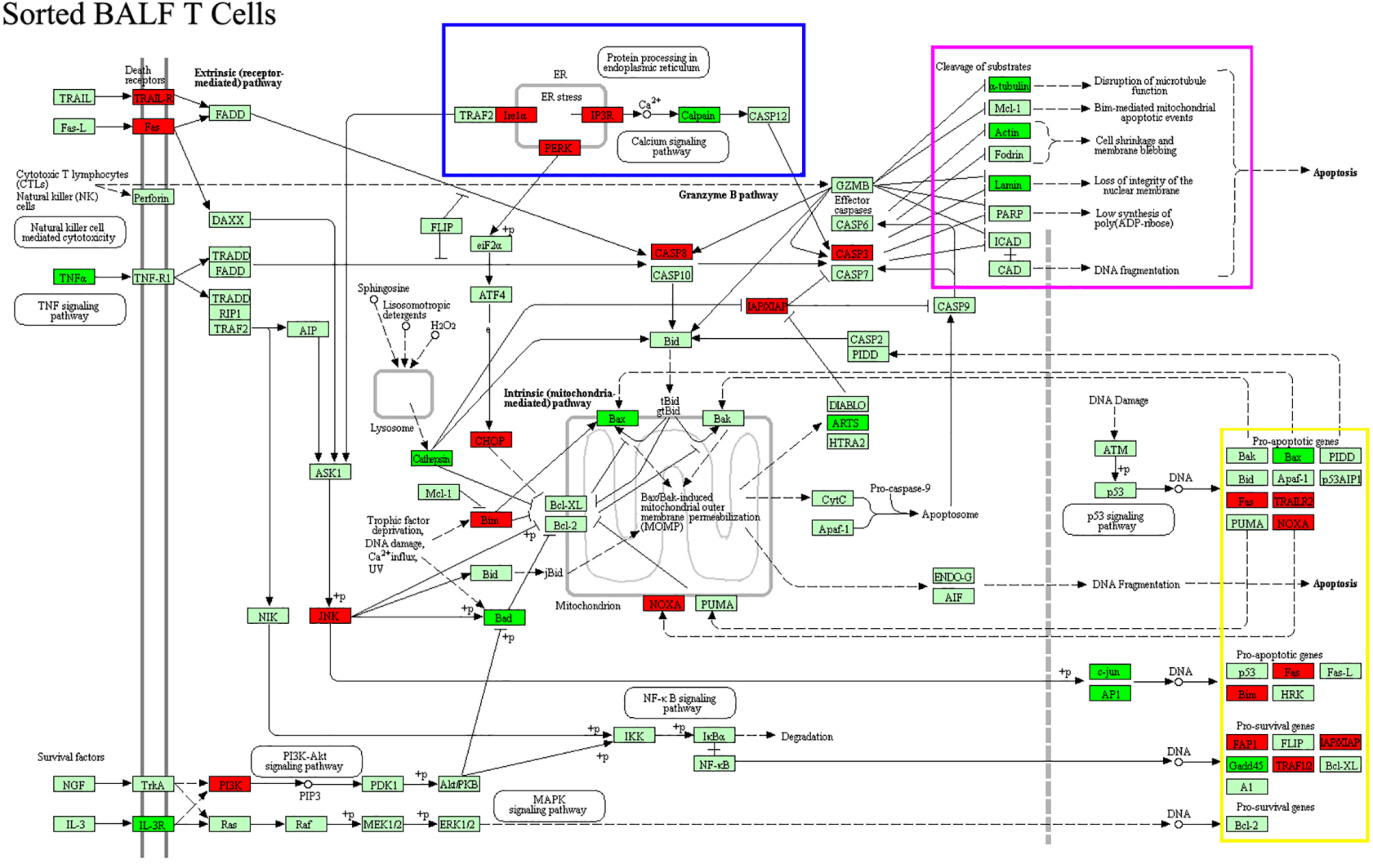
DEGs of apoptotic genes in patient-sorted BALF T cells generated from the comparison with sorted BALF T cells in FB controls were processed with KEGG for apoptosis pathway analysis. Upregulated genes were labeled with red color, downregulated green. Cluster of DEGs with common function that is exclusive in patient-sorted T cells are boxed with different colors. DEGs of ER stress are boxed with blue. Substrates of cleavage, such as cell skeleton genes, are boxed with purple. Proapoptotic genes for DNA damaging are boxed with yellow.

Cytokine gene analysis provided us with much intriguing information as we compared the DEGs derived from BALF cells with those derived from sorted T cells. Even though we did not observed an increase in IL-2 protein in patient aqueous BALF by Luminex, our mRNA microarray data indicated that IL-2 mRNA level was higher in sorted patient BALF T cells (**Figure 5B**). The fact that transcriptional level of IL-8 was increased in patient BALF cells but not in sorted BALF T cells suggested that IL-8 was likely released from the non-T-cell compartments (**Figure 5A**). Conversely, the transcriptional level of IFN-γ was shown increased only in sorted patient BALF T cells but not in BALF cells. In addition, total BALF cells but not sorted BALF T cells from patients displayed a higher IL-6 receptor (IL-6R) expression. This implied that cell compartments of non-T cells were possibly more sensitive to IL-6 signal (**Figures 5A, B**). OSM, a regulator for IL-6 production was highly expressed in patient BALF cells but decreased in sorted BALF T cells. In MPP patients, an increased expression level in IL-18R and IL-18RAP was found in both unpurified BALF cells and sorted BALF T cells (**Figures 5A, B**). This implied that IL-18 signaling could have played an indispensable role fighting against Mp infection in airway. Meanwhile, IL-16 and IL-26, two of proinflammatory factors involved in T-cell activation exclusively expressed in a higher level in sorted patient BALF CD3+ T cells (**Figure 5B**). Since GO analysis indicated a high ranking on apoptosis pathway based on the DEGs from sorted T cells, we compared the changes in apoptotic genes in both unpurified BALF cells and sorted T cells. The mRNA microarray revealed a very distinct DEGs in apoptotic genes in sorted T cells (**Figure 6**) in comparison with unpurified BALF cells (**Supplementary Figure S2**). Compared with respective controls, the sorted T cells in patients exhibited a change implicated with more apoptotic genes than unpurified BALF cells. KEGG analysis revealed that in sorted patient BALF T cells, DEGs comprised with those implicated in ER-stress/calcium signaling pathway and intrinsic mitochondria-mediated pathway (**Figure 6**). It is readily to notice that genes encoding proteins of cell skeleton, such as α-tubulin, actin and lamin, were greatly reduced in sorted patient BALF T cells. This data suggested that T-cell was subjected to a breakdown of cell integrity. In addition, those genes mediating DNA damage were exclusively upregulated in patient sorted BALF T cells (**Figure 6**; **Supplementary Figure S2**). Taken together, these results indicated a much stronger apoptotic activity was initialized in patient BALF T cells.

**Figure 7:**
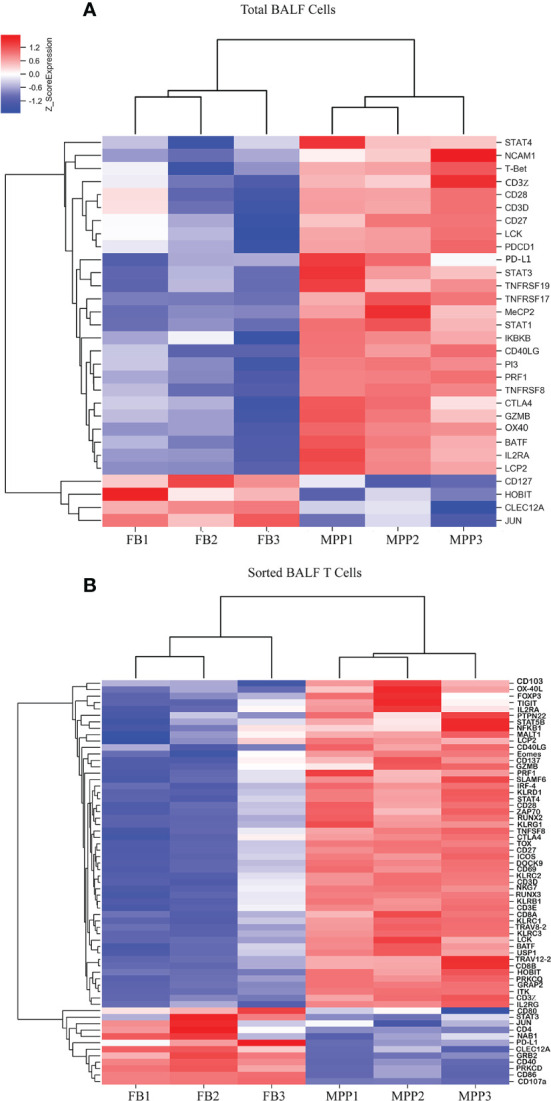
DEGs of T-cell activation and exhaustion derived from microarray data are represented as heat maps. Colors indicate relative expression levels. The gradient color from red to blue means expression of the listed gene from upregulation to downregulation. Gene names of DEGs are listed on the right. Similar pattern of expression are clustered closer with linked line. **(A)** The heat map for DEGs of T-cell activation and exhaustion gene in BALF cells. Each sample represents BALF cells from an individual patient or FB control. **(B)** The heat map for DEGs of T-cell activation and exhaustion in sorted CD3+ T cells. Each sample represents sorted CD3+ T cells from an individual patient or FB control.

Our mRNA microarray data showed that several lymphocyte activation markers were upregulated in unpurified patient BALF sample (**Figure 7A**). However, when sorted BALF T cells were compared, more upregulated T-cell activation markers emerged as DEGs (**Figure 7B**). In other words, more activation markers exclusively upregulated in T cells were disclosed with enhanced resolution through mRNA microarray and cell sorting, before which the transcriptional upregulation in T cells was offset by the changes in other BALF cells. Beside those upregulated DEGs responsible for T-cell activation being shared in unpurified BALF cells, gene expressions of IL-2Rα (CD25), IL-2Rγ (CD132),CD69,41BB,OX-40L, GITR, CD3ζ, PI3K, ZAP70, GADS, ITK, PKCθ, MALT1, KLRC1, KLRC2, KLRD1, DOCK9, NKG7, and STAT4 were remarkably upregulated in sorted patient BALF T cells (**Figure 7B**). Furthermore, it was obvious that patients BALF T cells displayed an expression profile pinpointed to the cell exhaustion as those typical exhaustion markers such as TIGIT, Eomes, TOX, KLRG1, ICOS, CTLA4, BATF, HOBIT, and FOXP3 were highly upregulated in sorted patient BALF T cells (**Figure 7B**) but not in unpurified BALF cells (**Figure 7A**). These results indicated that in inflammatory airway of MPP patients overactivated T cells were tuned into cell exhaustion in response to Mp infection.

## Discussion

Information for the immune response to Mp infection was mainly from studies on patient BALF. Flow cytometry analysis from previous researches revealed that T cells are one of the major cell populations in BALF. We studied on BALF from acute phase of severe MPP patients and found their major immune cell populations were T cells in memory phenotype (CD45RO+). The dominant CD4+ subpopulation in patient BALF T cells implies a strong CD4+ T-cell-mediated T helper effect in the inflammatory airway, differing the immune reaction against Mp infection from other pathogens such as virus. In our study, T cells in patients BALF expressed augmented levels of HLA-DR and CD25, which is in accordance with the previous study (11). Our *in vitro* BALF T-cell culture demonstrated that upon TCR ligation, patient BALF T cells released more IFN-γ and IL-2. This indicated that BALF T cells were sensitized for activation following Mp infection.

Measuring the serum and BALF cytokines/chemokines concentrations in parallel has two merits. Firstly, cytokines/chemokines being found change in both BALF and serum must be closely related to the disease conditions. Secondly, those only changed in BALF must be an immune response particularly belonging with the local inflammatory airway. Indeed, in our study, the IL-1β, IL-8, and TNF-α increased in both patient BALF and serum. These cytokines have been well documented for their drastic change in MPP patients (2, 12). IL-6, in our hand, showed a decreased expression in patient BALF but an increase in serum. The discrepancy between BALF and serum concentration could be a result from a stronger usage and sequester of IL-6 in the inflammatory airway.

The fact IFN-γ, RANTES (CCL5), and MCP1 increased while IFN-α2 slightly decreased only in patient BALF indicated these cytokines participated in responses restricted to the local airway instead of a systemic immune reaction. Interestingly, without a difference between patients and control, serum RANTES concentration presented with a surprisingly higher level than that in BALF. It is known that only highly ordered oligomer of RNATES possesses cell recruiting activity to the inflammatory sites. In physiological condition, high concentration of RANTES tend to aggregate with a muted activity. It is very likely serum RANTES in high concentration is polymerized and becomes indifferent to the environment changes in MPP patients (13, 14). By contrast, IL-10, IL-15, and IL-4 were found to increase in circulation but not in BALF, even though IL-10 was in a marginally higher concentration in patient BALF than in control.

In our study, mRNA microarray combined with cell sorting facilitated us to discern clearly how does these cytokines/chemokines expression being regulated and from where are they possibly derived. Our result revealed a distinctive expression profile of chemokines between total BALF cells and sorted BALF T cells in MPP patients. A bulk of chemokines were tremendously upregulated in patient BALF indicating an active chemotaxis in patient airway. By contrast, very limited number of chemokines in sorted T cells from patient BALF were upregulated. This implied that most of the chemotaxis forces in patient airway were derived from cell compartments other than T-cell population. Interestingly, several genes such as XCL1, XCL2, CCL5, CCR2, and CCR6 were exclusively upregulated in patient BALF T cells. XCL1 and XCL2 are two closely related analog genes being specifically chemotactic for T cells (15). CCR6 are preferentially expressed in immature dendritic cells and memory T cells (16). CCR2 regulates the expression of T-cell inflammatory cytokines and T-cell differentiation (17, 18). CCL5 are an effective molecule for CD4 T helper cell recruitment (19). These results suggest that BALF cells, most likely monocytes take the major responsibility for establishing the chemotactic microenvironment for the inflammatory airway, while T cells have unique chemokine expression profile for cell recruitment and migration to inflammatory sites. Our data revealed that the mRNA level of CXCL8 (IL-8) in patient was increased in BALF cells but not in sorted BALF T cells. This was congruent with the strongly increased IL-8 protein in BALF determined by Luminex implying that those cells other than T cells were the major contributor for the IL-8 production in inflammatory airway. The fact that RANTES (CCL5) mRNA level was preferentially higher in patient sorted T cells indicates that BALF T cells were most likely the major RANTES producer in BALF.

Our data showed that IFN-γ and IL-2 expressed at a significant higher level in sorted patient BALF T cells in related to their sorted T-cell counterpart in FB controls. This agreed with our *in vitro* stimulation experiment in which patients T cells released more IFN-γ and IL-2 than FB controls upon TCR ligation. Analysis of DEGs did not show a change of IL-6 expression in patient BALF cells. However, IL-6R and OSM, two molecules closely involved in IL-6 signaling, were found exclusively higher in patient BALF cells but not in sorted patient BALF T cells. This result implied that those non-T cells in patients’ airway could have possessed a better sensitivity in response to IL-6 signaling. One of interesting findings from our microarray data was that IL-18 was significantly downregulated in both BALF cells and sorted BALF T cells in patients. Conversely, two of IL-18 signaling receptors, IL-18R1 and IL-18RAP were greatly upregulated in total BALF cells and in sorted BALF T cells. We believe various types of BALF cells including T cells were strongly response to IL-18 signaling in the inflammatory airway in MPP patients. Considering the low expression of IL-18 in patient’s BALF cells, IL-18 was possibly derived from the insulted airway epithelial cells. It has been described that airway epithelial cells were induced to release IL-18 in other disease conditions such as asthma (20, 21). In the future it is necessary to determine the IL-18 level in airway epithelial cells of MPP patients.

Many activation markers in patient BALF cells were upregulated. This became more obvious when patient BALF T cells were sorted in which more activation markers were identified as DEGs between patients and FB controls. Furthermore, when BALF DEGs (generated from the comparison between patient BALF cells and FB BALF cells) and sorted DEGs (generated from the comparison between sorted patient BALF T cells and sorted FB BALF cells) were compared, many T-cell exhaustion markers prominently emerged in sorted DEGs. This clearly demonstrated that BALF T cells in acute phase of severe MPP patients were largely in a status of exhaustion. It has been known that exhausted T cells are at suboptimal function releasing less IL-2 and IFN-γ (22, 23). In the present study, we re-stimulated patient BALF T cells and found they produced higher IL-2 and IFN-γ than those from FB BALF T cells. This seemingly incongruence was not difficult to understand because patients BALF cells contained more sensitized T cells than those in FB control BALF. In other words, exhausted patient BALF T cells were at a suboptimal condition with a reduced IL-2 and IFN-γ-producing ability, yet still better than those non activated T cells in FB controls. Indeed, when we directly stained the cell surface exhaustion markers, we found TIM-3 and TIGIT were greatly upregulated in patient BALF CD3+ T cells. This surface protein level determination reinforced our findings in microarray assay for the mRNA expression of exhaustion genes.

We boldly believed that the overactivated T cells in patient BALF progressed into apoptotic course. Indeed, our microchip result revealed that in sorted patient BALF T cells, many genes related to cell machinery such as ER stress, mitochondria respiratory function, DNA damaging, and cytoskeleton had been tuned to the direction of cell death. In our direct staining methods, annexin V staining for the exposure of phosphatidylserine from the destructed cell membrane suggested that BALF CD3+ T cells in MPP patients had not progressed to the stage where cell membrane integrity has been destroyed. However, compared with FB control BALF CD3+ T cells, the JC-1 staining indicated that a change in mitochondrial membrane potential had been initialized in MPP BALF T cells. Whether cell death of T cells is a consequence from the convalescence of airway damage along with the attenuation of inflammation requires more investigations.

A variety of cells play as proinflammatory mediators in airway during Mp infection including but not limited to neutrophils, macrophages, and submucosal residential cells. While we emphasized the T-cell function in the inflammatory airway, we have to recognize that the inflammation is a sequelae from the complex interplay of multiple mediators. For example, it is important to delineate how does the damaged airway epithelia cell feedback to the proinflammatory microenvironment. Our data suggested that some important mediators, such as IL-8 and IL-1β, were not derived from T cells. However, it is impossible to infer from our study where did they come from without identifying the behavior of other cells. Meanwhile, our main evidence of the particular DEGs in patient BALF T cells was from mRNA microarrays. Differential protein expression would reflect more directly for the unique function and behavior of the BALF T cells in MPP patients. Thus, proteomic method and even single-cell sequencing would be better solutions for a deeper understanding. With enough resolution, these methods may provide a more intriguing picture of intertwined airway inflammation events. In the present study, we strictly chose the MPP patients at acute phase. We were not able to judge how much the behavior of T cells in our data could represent the situation in mild MPPs or at recovery stages. In the future, comparing the T-cell behavior in different MPP stages would harness identifying the true trigger for T-cell overactivation.

## Data Availability Statement

The datasets presented in this study can be found in online repositories. The names of the repository/repositories and accession number(s) can be found in the article/**Supplementary Material**.

## Ethics Statement

The studies involving human participants were reviewed and approved by the Ethics Committee of Beijing Children’s Hospital and the Ethics Committee of The Children’s Hospital Affiliated to the Capital Institute of Pediatrics. Written informed consent to participate in this study was provided by the participants’ legal guardian/next of kin.

## Author Contributions

XC and JG designed the project. XC, FL, XW, HZ, WM, BZ, XK, and AJ preformed the experiments. XC, JG, AJ, and SZ analyzed the data and wrote the manuscript. All authors contributed to the article and approved the submitted version.

## Funding

This study was supported by an intramural startup grant awarded to JG.

## Conflict of Interest

The authors declare that the research was conducted in the absence of any commercial or financial relationships that could be construed as a potential conflict of interest.

## Publisher’s Note

All claims expressed in this article are solely those of the authors and do not necessarily represent those of their affiliated organizations, or those of the publisher, the editors and the reviewers. Any product that may be evaluated in this article, or claim that may be made by its manufacturer, is not guaranteed or endorsed by the publisher.

## Supplementary Material

Supplementary Figure 1 The major cell subpopulations in BALF were analyzed with flow cytometry. **(A)** BALF cells from patients (MPP) and controls (FB) were stained with indicated flourochrome-conjugated antibodies labeled in the X and Y axis. The gating strategy was shown. The representative flow cytometry data were shown as pseudocolor dots. **(B)** The percentage of each cell subpopulations in BALF or in Blood (upper right panel only) were represented as dot plot with a mean ± SE. Statistics were done with Student *t*-test. NS, No significant difference. ****p* < 0.001. Each dot represents a sample from an individual patient or FB control.

Supplementary Figure 2 DEGs of apoptotic genes in patient total BALF cells generated from the comparison with total BALF cells in FB controls were processed with KEGG for apoptosis pathway analysis. Upregulated genes were labelled with red color, downregulated green.
