# Supplementary figures and images for: Exhausted and Apoptotic BALF T Cells in Proinflammatory Airway Milieu at Acute Phase of Severe Mycoplasma Pneumoniae Pneumonia in Children
Source: Front Immunol. 2022 Jan 17;12:760488. doi: 10.3389/fimmu.2021.760488 (PMC8801936; doi:10.3389/fimmu.2021.760488)

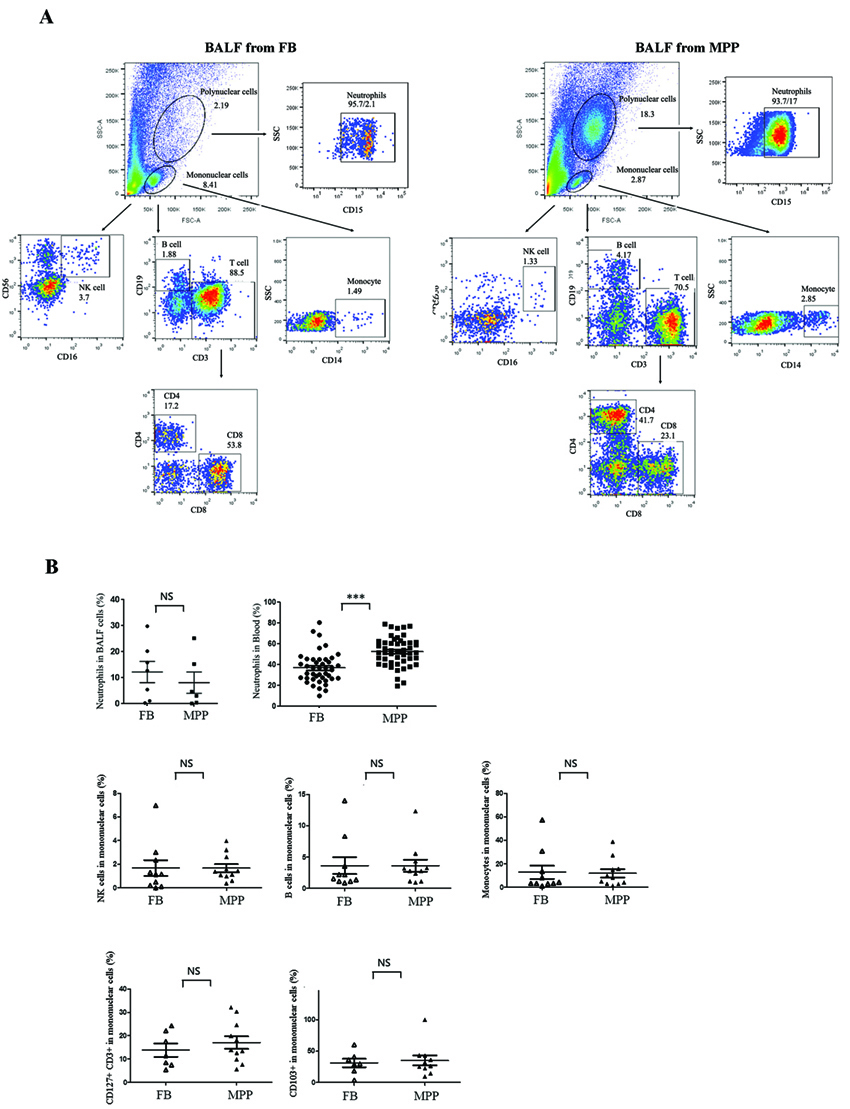

Supplement: Supplementary Figure 1 — The major cell subpopulations in BALF were analyzed with flow cytometry. (A) BALF cells from patients (MPP) and controls (FB) were stained with indicated flourochrome-conjugated antibodies labeled in the X and Y axis. The gating strategy was shown. The representative flow cytometry data were shown as pseudocolor dots. (B) The percentage of each cell subpopulations in BALF or in Blood (upper right panel only) were represented as dot plot with a mean ± SE. Statistics were done with Student t-test. NS, No significant difference. ***p < 0.001. Each dot represents a sample from an individual patient or FB control. [file Image_1.tif]

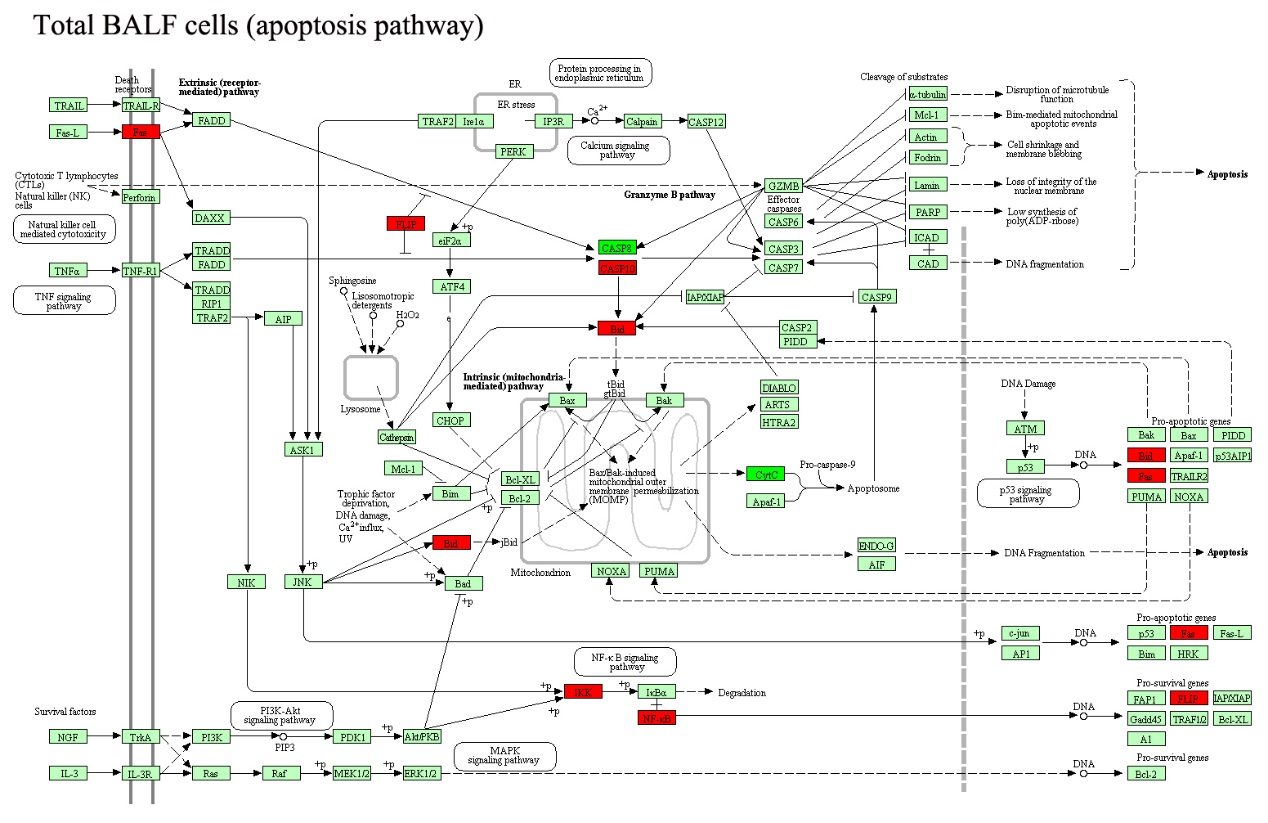

Supplement: Supplementary Figure 2 — DEGs of apoptotic genes in patient total BALF cells generated from the comparison with total BALF cells in FB controls were processed with KEGG for apoptosis pathway analysis. Upregulated genes were labelled with red color, downregulated green. [file Image_2.tif]
